# The Effect of DREADD Activation of Leptin Receptor Positive Neurons in the Nucleus of the Solitary Tract on Sleep Disordered Breathing

**DOI:** 10.3390/ijms22136742

**Published:** 2021-06-23

**Authors:** Mateus R. Amorim, Olga Dergacheva, Thomaz Fleury-Curado, Huy Pho, Carla Freire, David Mendelowitz, Luiz G. S. Branco, Vsevolod Y. Polotsky

**Affiliations:** 1 Division of Pulmonary and Critical Care Medicine, Department of Medicine, Johns Hopkins University School of Medicine, Baltimore, MD 21224, USA; tfleury2@jh.edu (T.F.-C.); hpho1@jhmi.edu (H.P.); cfreire2@jhmi.edu (C.F.); 2 Dental School of Ribeirão Preto, University of São Paulo, Ribeirão Preto, São Paulo 14040-904, Brazil; branco@forp.usp.br; 3 Department of Pharmacology and Physiology, George Washington University, Washington, DC 20037, USA; olgad@gwu.edu (O.D.); dmendel@gwu.edu (D.M.)

**Keywords:** obstructive sleep apnea, chemogenetics, upper airway dysfunction

## Abstract

Obstructive sleep apnea (OSA) is recurrent obstruction of the upper airway due to the loss of upper airway muscle tone during sleep. OSA is highly prevalent, especially in obesity. There is no pharmacotherapy for OSA. Previous studies have demonstrated the role of leptin, an adipose-tissue-produced hormone, as a potent respiratory stimulant. Leptin signaling via a long functional isoform of leptin receptor, LEPR^b^, in the nucleus of the solitary tract (NTS), has been implicated in control of breathing. We hypothesized that leptin acts on LEPR^b^ positive neurons in the NTS to increase ventilation and maintain upper airway patency during sleep in obese mice. We expressed designer receptors exclusively activated by designer drugs (DREADD) selectively in the LEPR^b^ positive neurons of the NTS of *Lepr^b^*-Cre-GFP mice with diet-induced obesity (DIO) and examined the effect of DREADD ligand, J60, on tongue muscle activity and breathing during sleep. J60 was a potent activator of LEPR^b^ positive NTS neurons, but did not stimulate breathing or upper airway muscles during NREM and REM sleep. We conclude that, in DIO mice, the stimulating effects of leptin on breathing during sleep are independent of LEPR^b^ signaling in the NTS.

## 1. Introduction

Obstructive sleep apnea (OSA) is a highly prevalent condition observed in approximately 9–45% of adult men and women [1,2], and in more than 50% of obese individuals [1,3,4,5,6]. OSA is defined as recurrent closure of the upper airway during sleep due to loss of upper airway muscle tone [7]. OSA is a major cause of morbidity and mortality in the Western World [8,9] and contributes significantly to the development and progression of neurocognitive, metabolic, cardiovascular, and oncologic diseases [10,11,12]. Up-regulation of oxygen-sensitive α-subunit of hypoxia-inducible factor 1 may contribute to metabolic disorders in OSA [12]. Continuous positive airway pressure is the first-line treatment modality for OSA, which improves clinical symptoms, quality of life, and gas exchange, but it is poorly tolerated by a large proportion of patients [13]. The absence of effective pharmacotherapy and adverse effects of traditional therapeutic approaches in OSA patients require rodent models to understand the neural mechanisms regulating the control of breathing and upper airway patency in OSA [14,15,16,17,18,19,20].

Our efforts have focused on leptin, an adipocyte-produced hormone, which suppresses appetite, increases metabolic rate [21,22,23], and acts as a powerful stimulant of breathing [24]. Leptin signaling occurs via the long isoform of the leptin receptor, LEPR^b^ [25], which is ubiquitous in many areas of the brainstem [26]. Leptin-deficient *ob/ob* mice hypoventilate during sleep and have elevated arterial CO_2_ (PaCO_2_), similar to human obesity hypoventilation syndrome (OHS) [24]. Recent studies have shown that *ob/ob* and leptin-resistant mice with diet-induced obesity (DIO) have increased upper airway collapsibility and develop inspiratory airflow limitation and OSA as well as OHS [14,27]. Enhanced plasma levels of leptin are commonly observed in obesity [28,29], but obese individuals are prone to develop resistance to the physiological effects of leptin. Intranasal leptin circumvented the blood–brain barrier and abolished OSA and OHS in obese mice [17]. Hence, LEPR^b^ signaling within the brain is an important target in OSA and OHS, but components related to the respiratory effects of leptin remain unknown.

The nucleus of the solitary tract [30,31,32,33], retrotrapezoid nucleus [34,35], and dorsomedial hypothalamus [19] are brain areas that may be involved in the pathophysiology and the treatment of OHS and OSA. LEPR^b^ was detected specifically in cell bodies within the NTS [26], an important region of the brainstem that processes afferent visceral information and controls breathing [36]. Local administration of leptin in the NTS increases pulmonary ventilation, respiratory volume [30], as well as ventilatory responses to hypercapnia in anesthetized rats [31]. On the other hand, optogenetic stimulation of LEPR^b^ positive NTS neurons increases phrenic nerve burst amplitude and transiently depresses phrenic nerve burst frequency [32].

However, to our knowledge, the role of LEPR^b^ positive neurons in the NTS in respiration and upper airway function during sleep has not been examined. We assess upper airway function during sleep by the presence of inspiratory flow limitation (IFL), an early inspiratory plateau in airflow at a maximum level (V_I_max) while effort continued to increase [16,37,38,39]. IFL is the cardinal feature of upper airway obstruction during sleep in humans with OSA [40,41,42,43]. Our approach allows us to characterize non-flow limited breathing, an integral measure of metabolism and control of breathing, and upper airway obstruction differentially during sleep. In the present study, we hypothesized that leptin acts on LEPR^b^ positive neurons in the NTS to increase ventilation and maintain upper airway patency during sleep in obese mice. To test this hypothesis, we expressed designer receptors exclusively activated by designer drugs (DREADD) exclusively in LEPR^b^ positive NTS neurons of C57BL6/J mice with diet-induced obesity, and performed sleep studies and genioglossus activity recordings after treatment with a specific DREADD ligand J60 [44,45] vs. saline using a crossover study design.

## 2. Results

### 2.1. Histology and Electrophysiology

**Figure 1:**
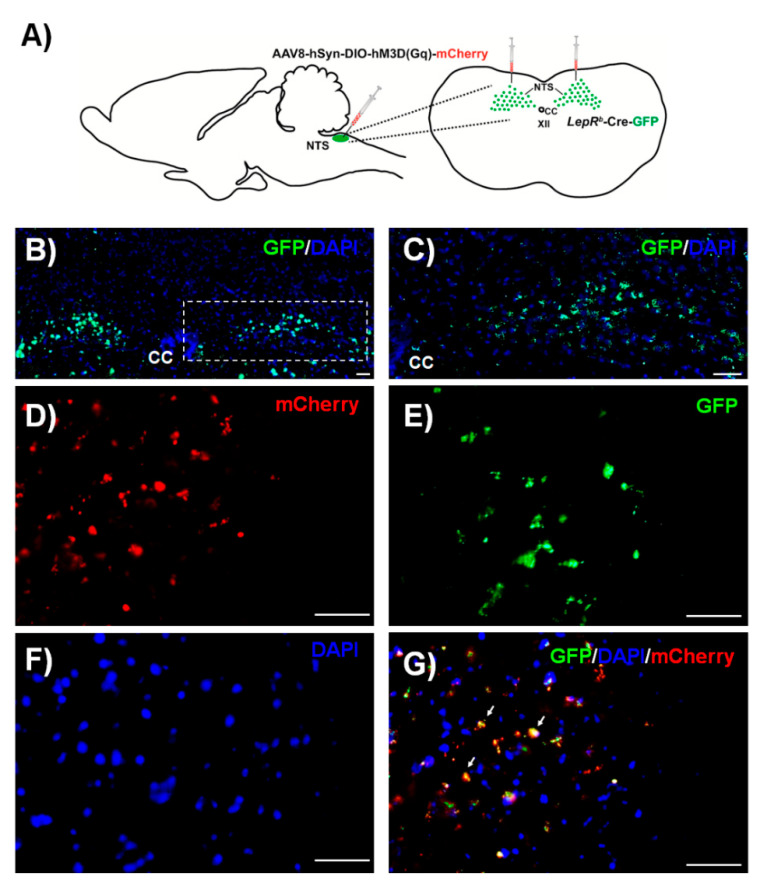
(**A**) Experimental design and injection sites of AAV8-hSyn-DIO-hM3D(Gq)-mCherry in the nucleus of the solitary tract (NTS) region. Localization of LEPR^b^ positive neurons (green) in the NTS (**B**). The same, at a higher power (**C**). Transfection of DREADD (red in (**D**)) in the LEPR^b^ positive neurons of the (green in (**E**)); DAPI (4′,6-diamidino-2-phenylindole) represents nuclei (blue in (**F**)). The orange color resulting from the red and green colors overlapping demonstrates DREADD expression in the LEPR^b^ positive neurons (**G**). Scale bars, 50 µm. Arrows represent the co-localization of DREADD and GFP.

**Figure 2:**
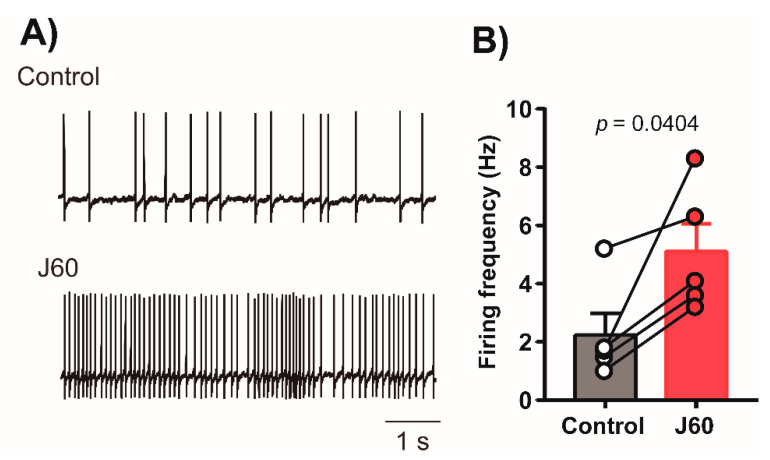
In-vitro activation of NTS neurons that contain DREADD in LEPR^b^ positive neurons. (**A**) Representative tracing demonstrates an increase in action potential firing of the DREADD-containing NTS neuron, recorded in current-clamp configuration before and after J60 application. (**B**) The summary data from 5 neurons illustrate a significant increase in firing activity of NTS neurons post J60 application.

LEPR^b^ positive neurons were abundantly expressed in the NTS. DREADD were successfully deployed in these neurons 4–6 weeks after AAV8-hSyn-DIO-hM3D(Gq)-mCherry administration (Figure 1). Recordings in acute brainstem slices confirmed that the DREADD ligand J60 excited LEPR^b^ positive NTS neurons (Control: 2.2 ± 0.8 vs. 5.1 ± 1.0 Hz, Figure 2).

### 2.2. Sleep Studies

**Figure 3:**
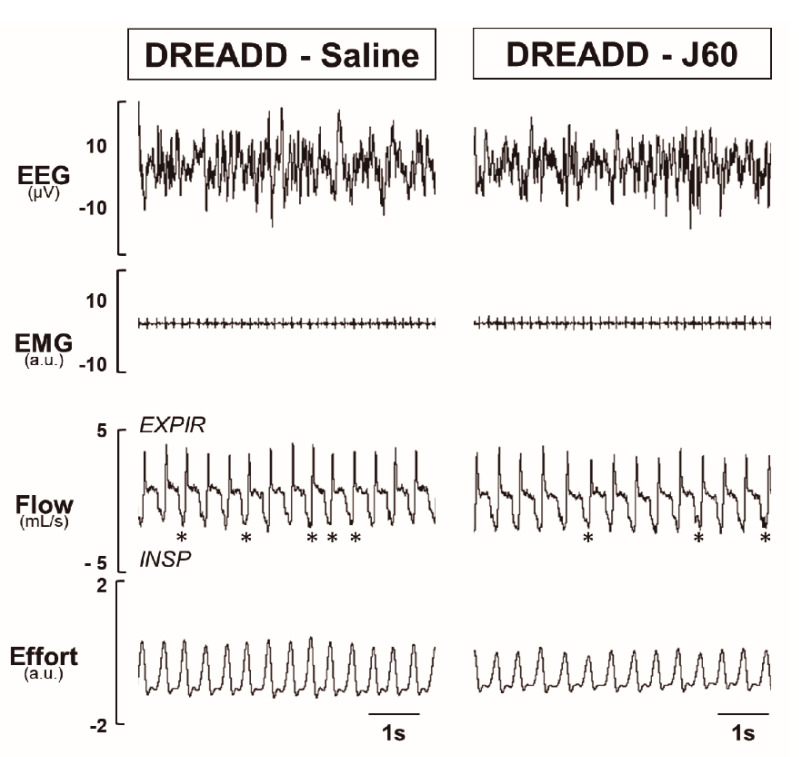
Sleep recordings. Representative traces of NREM sleep recordings in the same mouse expressing designer receptor exclusively activated by designer drug (DREADD) in the NTS after treatment with saline versus J60. * denotes breaths with inspiratory flow limitation.

**Figure 4:**
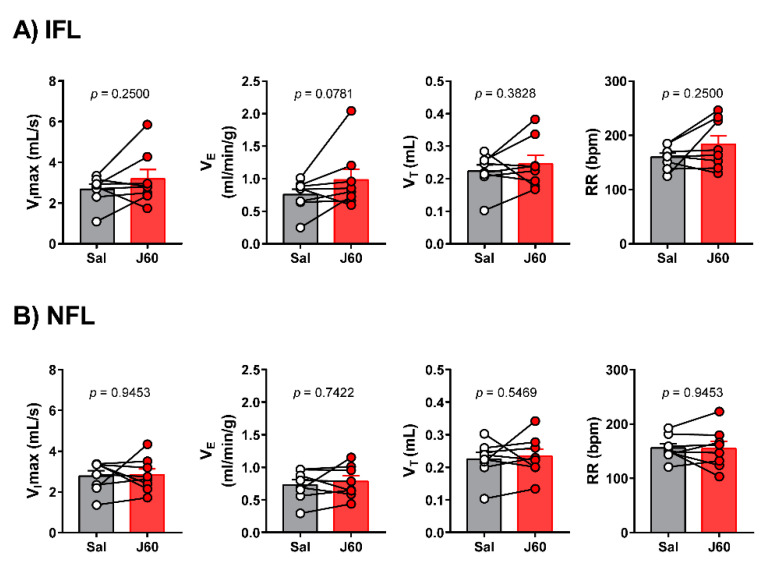
Individual and grouped data showing the effects of J60 ligand or saline on maximal inspiratory flow (V_I_max), minute ventilation (V_E_), tidal volume (V_T_) and respiratory rate (RR) during flow-limited (**A**) and nonflow-limited breathing (**B**) in nonrapid eye movement (NREM) sleep.

To determine if DREADD activation in the LEPR^b^ positive neurons alleviates upper airway obstruction and up-regulates the control of breathing during sleep, we used our model of sleep-disordered breathing in diet-induced obese mice. Figure 3 illustrates the effect of J60 ligand on breathing in NREM sleep. The representative recordings of NREM sleep show that J60 did not stimulate breathing (non-flow limited breaths) or upper airway function (IFL breathing) compared to saline (Figure 3). Our data show that activation of LEPR^b^ positive neurons in NTS did not affect any of the respiratory parameters. The respiratory indexes after saline and J60 treatments respectively were as follows: V_I_max (IFL breathing: 2.66 ± 0.25 vs. 3.17 ± 0.46 mL·s^−1^; non-flow limited breathing: 2.78 ± 0.26 vs. 2.84 ± 0.29 mL·s^−1^), minute ventilation (V_E_, IFL: 0.75 ± 0.08 vs. 0.98 ± 0.17 mL/min/g; non-flow limited breathing: 0.73 ± 0.08 vs. 0.78 ± 0.09 mL/min/g), tidal volume (V_T_, IFL: 0.22 ± 0.02 vs. 0.25 ± 0.03 mL; non-flow limited breathing: 0.22 ± 0.02 vs. 0.23 ± 0.02 mL) and respiratory rate (RR, IFL: 160 ± 7 vs. 183 ± 16 bpm; non-flow limited breathing: 156 ± 8 vs. 155 ± 13 bpm); *p* > 0.05 for all (Figure 4).

**Figure 5:**
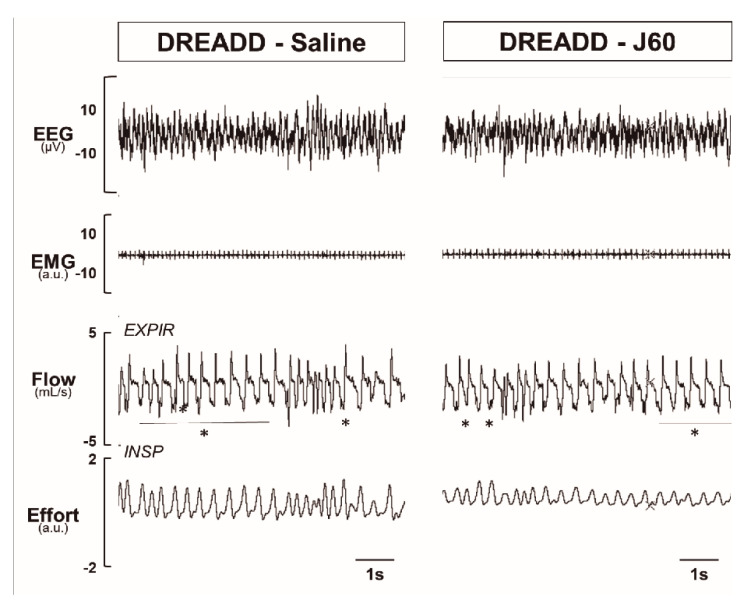
Sleep recordings. Representative traces of REM sleep recordings in the same mouse expressing DREADD in the NTS after treatment with saline versus J60. * denotes breaths with inspiratory flow limitation.

**Figure 6:**
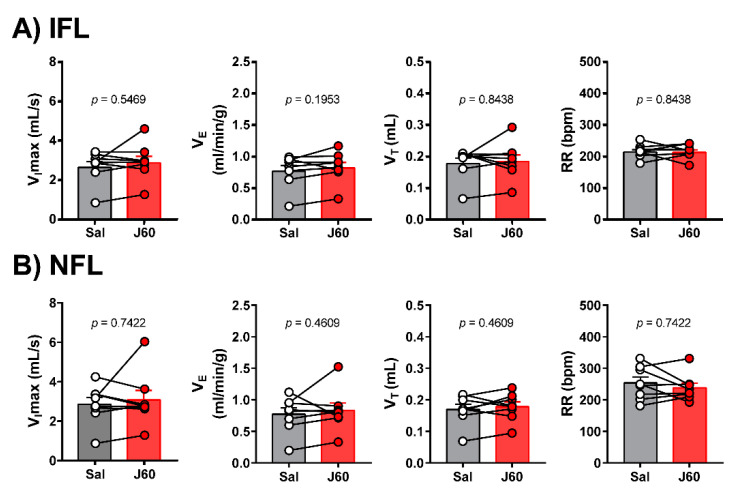
Individual and grouped data showing the effects of J60 ligand or saline on maximal inspiratory flow (VImax), minute ventilation (V_E_), tidal volume (V_T_), and respiratory rate (RR) during flow-limited (**A**) and nonflow-limited breathing (**B**) in rapid eye movement (REM) sleep.

REM sleep and breathing were also recorded by barometric plethysmography. A representative polysomnography in REM sleep demonstrates that J60 ligand did not affect breathing or upper airway function in comparison with saline (Figure 5). The analysis of breathing during REM sleep showed no significant difference between saline and J60, respectively, for V_I_max (IFL: 2.68 ± 0.29 vs. 2.91 ± 0.33 mL·s^−1^; non-flow limited breathing: 2.86 ± 0.35 vs. 3.08 ± 0.48 mL·s^−1^), V_E_ (IFL: 0.78 ± 0.09 vs. 0.83 ± 0.09 mL/min/g; non-flow limited breathing: 0.77 ± 0.09 vs. 0.83 ± 0.12 mL/min/g), V_T_ (IFL: 0.18 ± 0.02 vs. 0.19 ± 0.02 mL; non-flow limited breathing: 0.17 ± 0.02 vs. 0.18 ± 0.02 mL) and RR (IFL: 216 ± 8 vs. 215 ± 8 bpm; non-flow limited breathing: 254 ± 19 vs. 238 ± 15); *p* > 0.05 for all, Figure 6.

### 2.3. Electromyography of the Genioglossus Muscle (EMG_GG_)

**Figure 7:**
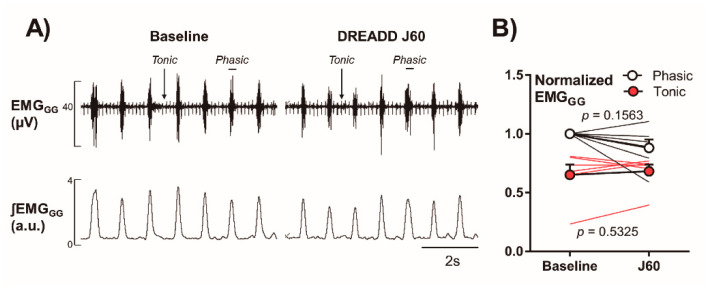
Genioglossus EMG recordings. (**A**) Representative genioglossal electromyography (EMG_GG_), moving average (ʃEMG_GG_), recorded at baseline (left) and after J60 administration (right) in a mouse expressing DREADD in the NTS. (**B**) Individual and grouped data showing the effects of J60 ligand on EMG_GG_ in DREADD-treated animals.

In six DREADD-infected mice who underwent EMG_GG_ testing after administration of J60 ligand, we documented that J60 had no effect on tonic or phasic activity as compared to baseline (Phasic: 1.00 ± 0.00 vs. 0.88 ± 0.07-fold; Tonic: 0.65 ± 0.09 vs. 0.68 ± 0.06, *p* > 0.05, Figure 7).

## 3. Discussion

The main finding of our study is that in the presence of J60 ligand, selective expression of AAV8 double floxed Gq-coupled hM3D DREADD in LEPR^b^ positive NTS neurons (Figure 1) did not stimulate breathing, upper airway function during NREM and REM sleep (Figure 3 and Figure 5), or tonic and phasic activity of GG muscle (Figure 7). Our positive control showed a robust increase in firing frequency of DREADD-containing NTS LEPR^b^ positive neurons (Figure 2). To our knowledge, this is the first study examining whether stimulation of LEPR^b^ positive neurons in NTS treats sleep disordered breathing.

### 3.1. The Effect of Activation of LEPR^b^ Positive NTS Neurons on Breathing during Sleep

NTS is a heterogeneous population of second order neurons in the dorsolateral medulla, extending from the caudal margin of the facial nucleus to the caudal margin of the pyramidal decussation [36,46]. NTS neurons process afferent visceral information from peripheral chemoreceptors in the carotid bodies (CB), and send synaptic contacts to different regions of the central nervous system involved in the respiratory control and central chemosensitivity [36,47]. In vitro experiments showed that NTS contains CO_2_-sensitive neurons that are putative central chemoreceptors [48]. Previous experiments from our group demonstrated that leptin-resistant obese mice hypoventilate during sleep and have significantly higher waking CO_2_ and a decrease in CO_2_ central chemosensitivity [16]. Functional isoform of leptin receptors (LEPR^b^s) was described in the NTS neurons [26,49]. Systemic administration of leptin up-regulated phospho-STAT3 immunoreactivity in the NTS [50], indicating that LEPR^b^ signaling in the NTS neurons may be implicated in increased CO_2_ chemosensitivity [31,51,52].

Leptin’s effects on the NTS neurons may merely transmit the peripheral action of the hormone in the carotid body (CB). Hypoxic and hypercapnic stimuli signal from the CB [53,54] to the dorsal vagal complex located in the NTS. Leptin acts on the CB, increasing hypoxic ventilatory response and this effect of leptin is abolished by CB denervation [55,56]. Leptin interacts with CB via receptor potential melastatin 7 (TRPM7) channel [57] and the up-regulation of leptin-TRPM7 axis in CB augments hypoxic ventilatory response hypertension. Future studies are needed to investigate whether the LEPR^b^ positive CB cells project to LEPR^b^ positive NTS neurons, and if this pathway is dependent on TRPM7 channel activity to up-regulate breathing in obese mice [58]. Intranasal administration of leptin did not affect leptin plasma levels [17], but delivered leptin to the medulla [59] and reversed OSA in leptin-resistant mice, which suggests that respiratory effects of leptin are independent of peripheral signaling in the CB and the CB-NTS axis.

Inyushkin et al., (2009) [30] measured respiratory responses to microinjections of leptin into the ventrolateral NTS and reported that single administration of leptin into the NTS of anesthetized rats acutely increased the EMG activity of the inspiratory muscles and central chemosensitivity in a concentration-dependent matter [31]. A recent study by Do et al. (2020) [32], using state-of-art approaches, demonstrated that optogenetic stimulation of ChR2-expressing LEPR^b^ positive NTS neurons in anesthetized and mechanically ventilated mice transiently increased the burst amplitude and depressed burst frequency of the phrenic nerve. Altogether, previous studies are consistent with the notion that leptin upregulated ventilatory control acting in the hindbrain, possibly in the NTS.

Contrary to our main hypothesis that leptin exerts its beneficial effect on sleep disordered breathing in the NTS, our data are consistent with the notion that even though the J60 ligand was a potent activator of LEPR^b^ positive NTS neurons (Figure 2), DREADD activation of these neurons did not stimulate breathing during NREM and REM sleep. Reconciling our present study and previous investigations, we would like to emphasize several important methodological differences: (1) our in vivo approach in unanesthetized freely-behaving mice allows us to exclude artefacts of anesthesia and in vitro studies, which cannot account for cross-talk between leptin responsive neurons in different areas of the brain; (2) our studies in a mouse model of obesity-induced sleep disordered breathing may yield different results from experiments in healthy leptin-sensitive lean animals; (3) prolonged respiratory recordings in sleeping animals eliminates behavioral effects and takes into account compensatory neural pathways, which may be activated in the LEPR^b^ positive NTS neurons; (4) a chemogenetic NTS-LEPR^b^ approach eliminates potential off-site effects of leptin microinjections; (5) leptin may exert competing excitatory and inhibitory effects on different populations of NTS neurons [32,60].

Overall, our data suggests that stimulation of LEPR^b^ positive NTS neurons does not stimulate non-flow limited ventilation during sleep. Non-flow limited breathing is breathing in the absence of upper airway obstruction, i.e., sleep apnea, and reflects both metabolism and control of breathing. Thus, we have demonstrated that stimulation of LEPR^b^ positive NTS neurons does not stimulate control of breathing during sleep and does not relieve OHS.

### 3.2. The Effect of Activation of LEPR^b^ Positive NTS Neurons in the Upper Airway Pattency

The main feature of OSA is the periodic upper airway obstruction during sleep [61], which is especially common in obesity due to anatomic predisposition and elevated mechanical load on the upper airway [62,63]. Considerable effort has been dedicated to understanding of the complex tongue neuromotor control mechanisms alleviating OSA [45,64]. The genioglossus muscle, an extrinsic tongue muscle responsible for protrusion and depression of the tongue, plays a key role in the maintenance of pharyngeal patency during sleep [64]. In the present study, we took advantage of our model of sleep-disordered breathing in obese mice, in which the upper airway physiology is very similar to humans, to test the hypothesis that DREADD activation of LEPR^b^ positive NTS neurons relieves upper airway patency in diet-induced obese mice.

Our data also showed that J60 did not improve IFL, i.e., upper airway patency, during NREM and REM sleep, and did not increase genioglossus muscle activity. Taken together, these data suggest that activation of LEPR^b^ positive neurons in the NTS does not stimulate upper airway muscles during NREM and REM sleep. We have previously reported that leptin activates the central nervous system relieving upper airway obstruction during sleep, in leptin-resistant obese mice [17]. We have also reported that leptin relieves upper airway obstruction during sleep, acting in the forebrain but not in the hindbrain, where NTS is located [14]. Nevertheless, LEPR^b^ NTS neurons may regulate upper airway patency, since the NTS is synaptically connected to the hypoglossal nucleus [17]. Our current study resolves this controversy by showing that stimulation of the LEPR^b^ positive NTS neurons does not treat OSA.

The study had limitations. First, we used younger mice in electrophysiological experiments with in vitro recordings compared in vivo studies. [15,45]. Second, although we have shown that nearly all LEPR^b^ positive NTS neurons were transfected with DREADD, the number of DREADD receptors may have been limited. The absence of a stimulating effect of J60 ligand on breathing or upper airway muscles sleep-stages may be related to inadequate efficiency of the transfection required to translate to physiological effects in the whole animal.

In conclusion, our study provided evidence that, in DIO mice, the stimulating effects of leptin on breathing during sleep are independent of LEPR^b^ signaling in the NTS.

## 4. Materials and Methods

### 4.1. Animals and Ethical Approval

All experimental protocols were approved by the Johns Hopkins University Animal Use and Care Committee (Protocol # MO19M191 approved on 06/10/2019). In total, eight adult male *Lepr^b^*-GFP mice expressing GFP in LEPR^b^ cells were used in the experimental protocols. *Lepr^b^*-GFP mice were generated by crossing *Lepr^b^*-Cre with GFP-floxed mice (B6.129-Gt(ROSA)26Sortm2Sho/J). Mice were fed with a high fat diet (TD 03584, Teklad WI, 5.4 kcal/g, 35.2% fat, 58.4% kcal from fat) for 12 weeks and used in our experiments at 20–30 weeks of age. Mice were housed at standard environmental conditions (24–26 °C in the 12-h light/dark cycle, 9 AM–9 PM lights on) and had free access to food and water, until the experiments started. For the in-vitro patch clamp experiments all animal procedures were approved by the George Washington University Institutional Animal Care and Use Committee, and were in compliance with the panel of Euthanasia of the American Veterinary Medical Association and the National Institutes of Health (NIH) Guide for the Care and Use of Laboratory Animals.

### 4.2. Viral Vector Administration

Mice were anesthetized with isoflurane for induction (2–3% in the closed chamber) and, after the absence of withdrawal reflex to a firm pinch of tail, they were set within the Kopf stereotaxic apparatus (model 963; Kopf Instruments, Tujunga, CA, USA) with mouse adapter VetEquip, and isoflurane vaporizer. Afterward, anesthesia was kept with 1–2% isoflurane. DREADD (AAV8-hSyn-DIO-hM3D(Gq)-mCherry, 4 × 10^12^ vg·mL^−1^, 100–200 nl, Addgene, Watertown, MA, USA) was delivered bilaterally using pre-pulled glass micropipettes (Sutter Instrument, Novato, CA, USA) to the NTS using the following stereotactic coordinates from the animal’s bregma: 7.60 to 7.80 mm anterior-posterior, 0.40 mm medial-lateral bilateral, and 4.80 to 5.00 mm dorso-ventral. The mice additionally received subcutaneous Buprenorphine (0.01 mg·kg^−1^) at the end of surgery to minimize distress.

### 4.3. Sleep Studies

Sleep studies were performed four to six weeks after DREADD administration in a modified plethysmography chamber, as previously described [19,37]. In a nutshell, under 1–2% isoflurane anesthesia mice underwent headmount (no. 8201, Pinnacle Technology, Lawrence, KS, USA) surgery with implantation of 4 electroencephalogram (EEG) electrode screws placed bilaterally in the frontal and parietal bones and 2 electromyography (EMG) leads placed in the nuchal muscles. After a one-week recuperation period, mice were set into a mice whole-body plethysmography (WBP, Buxco, Wilmington, NC, USA) chamber for acclimation. On the following day, tidal airflow, respiratory effort, and sleep–wake state were continuously recorded (~6 h) in the WBP chamber. Sleep studies were done after mice were treated with DREADD ligand J60 (0.1 mg/kg in 250 µL saline i.p.) or saline. The chamber was calibrated to allow high-fidelity tidal volume and airflow signals. Atmospheric pressure equilibrium was obtained by a slow leak and the chamber was humidified to 90% relative humidity. Positive and negative pressure sources in series with mass flow controllers and high-resistance elements created continuous airflow through the chamber. Conversion of the plethysmography chamber pressure signal to tidal volume was done using the Drorbaugh and Fenn equation. Mouse rectal temperature, chamber temperature, room temperature, and relative humidity, a known volume injection (1 mL), and the resultant WBP pressure deflection, as well as chamber gas constant, were used to calculate tidal volume signal from the WBP.

NREM sleep was characterized by high-amplitude, low frequency (~2 to 5 Hz) EEG waves with EMG activity considerably less than during wakefulness. REM sleep was characterized by low-amplitude, mixed frequency (~5 to 10 Hz) EEG waves with EMG amplitude either below or equal to that during NREM sleep.

A respiratory effort sensor bladder placed below the mouse transduced the mechanical pressure changes related to mouse breathing, whereas the reference bladder signal allowed for cancellation of the contaminating chamber pressure signal through the differential pressure transducer. All signals were digitized at 1000 Hz (sampling frequency per channel) and recorded in LabChart 7 Pro (Version 7.2, ADInstruments, Dunedin, NZ, USA).

Differentiation of the beginning and end of inspiration and expiration for subsequent calculations of timing and classification of the breath as flow limited and was done using custom software. Obstruction was identified by inspiratory flow limitation (IFL) characterized by an early plateau in inspiratory airflow at a maximum level (V_I_max) in the presence of increased effort. Respiratory analysis was performed separately during IFL and non-flow limited breathing during NREM and REM sleep.

EEG and EMG were used to score sleep–wake state in 5 s epochs and the respiratory signal was sampled from periods of stable NREM sleep all through the recording. Respiratory signals were analyzed from all REM sleep periods and from steady of NREM sleep sampled tested intermittently at 20-s extends each half an hour all through the entire recording time. All parameters were scored by one investigator, who was blinded to experimental conditions.

### 4.4. Electromyography of the Genioglossus Muscle (EMG_GG_)

EMG_GG_ recordings were done and analyzed as previously described [65]. 6–8 wks after DREADD administration, mice were deeply anesthetized with isoflurane (2–3%) and fixed in the supine position. A small ventral midcervical incision was performed along the submentum. Thereafter, isoflurane was held at 1–2% to maintain a respiratory rate at 1 Hz. Two wire hook electrodes (0.008 inches teflon-coated stainless steel wire, A-M Systems, Sequim, WA, USA) with bared ends (0.5 mm) were inserted in the GG muscle toward the base of the tongue using a 27 G needle to direct the intramuscular placement of the wire hooks. The hooks and the wires were sutured to the neck muscles to maintain placement. The EMG signal was amplified, filtered between 30 Hz and 1000 Hz using Bio Amplifier 8/35 (ADInstruments Inc. Colorado Springs, CO, USA), and digitized at a sampling rate of 1000 Hz in LabChart 7 Pro (Version 7.2, ADInstruments, Dunedin, NZ). In addition to the raw EMG signal, the moving average was obtained (100 ms time constant). The EMG_GG_ was rectified, and a 100 ms time constant was applied to compute the moving average (LabChart 7 Pro).

EMG_GG_ was recorded at baseline and after mice were treated with DREADD ligand J60 (0.1 mg/kg in 250 µL saline i.p.). For quantitative analysis, the tonic (expiratory) and peak phasic (inspiratory) components were measured for 10 randomly selected breaths 15 min before and after J60 injections. Tonic and phasic EMG_GG_ measurements were normalized and expressed as a percent of average peak phasic moving average at baseline.

### 4.5. Selective Expression and Activation of DREADD in NTS LEPR^b^ Positive Neurons

Selective expression of excitatory Designer Receptors Exclusively Activated by Designer Drugs (DREADD) was accomplished by microinjection of AAV8-hSyn-DIO-hM3(Gq)-mCherry into the NTS in *Lepr^b^*-Cre mice [B6.129(Cg)-Leprtm2(cre)Rck/J, JAX Stock #008320]. Mouse pups (4–7 days old) were anesthetized with hypothermia. Animals were mounted in a stereotactic apparatus with a neonatal adapter (Stoelting, Wood Dale, IL). The skull was exposed, and a small hole was made to position a pulled calibrated pipette (VWR, Radnor, PA, USA) containing viral vector. Viral vector (50 nL) was slowly injected at the following stereotactic coordinates for the NTS, from the bregma: −4.2 mm anterior-posterior, 0.2 mm medial-lateral, and 1.5 mm dorso-ventral. After surgery, buprenorphine was administered, and animals were monitored for 30 min and every 20 min thereafter until ambulatory.

On the day of experiment, animals (21–30 days old) were anesthetized with isoflurane, sacrificed, and glycerol-based artificial cerebrospinal fluid (aCSF) was perfused transcardially. Glycerol-based aCSF (4 °C) contained (in mM): 252 glycerol, 1.6 KCl, 1.2 NaH_2_PO_4_, 1.2 MgCl, 2.4 CaCl_2_, 26 NaHCO_3_, and 11 glucose. The brain was removed, and 300-μm-thick coronal slices of the medulla containing the NTS were obtained with a vibratome. The slices were then transferred to a solution of the following composition (in mM) 110 N-methyl-d-glucamine (NMDG), 2.5 KCl, 1.2 NaH_2_PO_4_, 25 NaHCO_3_, 25 glucose, 0.5 CaCl_2_, and 10 mM MgSO_4_ equilibrated with 95% O_2_–5% CO_2_ (pH 7.4) at 34 °C for 15 min. The slices were then transferred from NMDG-based aCSF to a recording chamber, which allowed perfusion (5–10 mL/min) of aCSF at room temperature (25 °C) containing (in mM) 125 NaCl, 3 KCl, 2 CaCl_2_, 26 NaHCO_3_, 5 glucose, and 5 HEPES equilibrated with 95% O_2_–5% CO_2_ (pH 7.4) for at least 30 min before experiments were conducted.

Individual neurons in the NTS were identified by mCherry fluorescence. These identified neurons were then imaged with differential interference contrast optics, infrared illumination, and infrared-sensitive video detection cameras. Patched pipettes (2.5–3.5 MΩ) were filled with a solution consisting of 135 mM K-gluconic acid, 10 mM HEPES, 10 mM EGTA, 1 mM CaCl_2_, and 1 mM MgCl_2_. Action potentials of NTS neurons were recorded in current clamp configuration with an Axopatch 200B and pClamp 9 software (Axon Instruments, San Jose, CA, USA). J60 (0.1 μM) was added to the perfusate to examine its effects on spontaneous action potential firing of DREADD expressing NTS neurons.

Analysis of firing activity of NTS neurons was performed using Mini Analysis (version 5.6.12; Synaptosoft, Decatur, GA, USA). Firing activity was quantified from a 1 min period prior to J60 application (control), and from 1-min period beginning immediately after J60 application.

### 4.6. Immunofluorescence

Expression of the AAV8-hSyn- DIO-hM3D(Gq)-mCherry viruses was confirmed by positive expression of mCherry in the LEPR^b^ positive neurons in the NTS. For the immunofluorescent analysis of the brain, mice were anesthetized with 1–2% isoflurane and promptly perfused transcardially using ice-cold phosphate-buffered saline (PBS; 0.01 M, pH 7.4) solution followed by 4% paraformaldehyde (PF) in 0.1 M PBS. Brains were removed, kept for 4 h in 4% PF and cryoprotected in 30% sucrose overnight at 4 °C. Afterward, tissues were embedded in Tissue-Tek O.C.T. (cat# 4583, Tissue Tek, Torrance, CA, USA), and frozen on dry ice. Coronal sections of the frozen brains were cut into 30 μm slices using a cryostat (HM 560, Thermo Scientific, Walldorf, Germany) and tissue was stored at −20 °C before further processing. Slides were kept at room temperature for 5 min and then rehydrated with PBS. Sections were covered with mounting medium with DAPI (4,6-diamidino-2-phenylindole; Vectashield, Vector Labs, Burlingame, CA, USA). Images from the different experimental groups were captured and then examined under a fluorescent microscope (Zeiss, Jena, Germany). Localization of DREADD in the brainstem was confirmed by visualization of mCherry expression. The following filters were used: for DAPI, excitation: 353 nm and emission: 465 nm; mCherry: excitation 650 nm and emission: 673 nm; and for GFP excitation 488 nm and emission: 509 nm.

### 4.7. Statistical Analysis

Statistical analysis was performed using Prism, version 7.03 (GraphPad Software Inc., San Diego, CA, USA). All of the data included in the present study were tested for normality using Shapiro–Wilk tests. Data that did not follow the normal distribution were analyzed using non-parametric statistics. To compare differences within groups we used paired Student’s T-test or Wilcoxon matched-pairs signed rank test. Data are expressed as the mean ± SEM. Significant difference was set at *p* < 0.05, although the exact *p* values are reported.

## Data Availability

The raw data that support the findings of this study are available from the corresponding authors upon request.
